# Supplementary material for: Strategies for the Site-Specific Decoration of DNA Origami Nanostructures with Functionally Intact Proteins
Source: ACS Nano. 2021 Aug 31;15(9):15057–68. doi: 10.1021/acsnano.1c05411 (PMC8482763; doi:10.1021/acsnano.1c05411)
Supplement: Supplementary file 1 — nn1c05411_si_001.pdf [file nn1c05411_si_001.pdf]

STRATEGIES FOR THE SITE-SPECIFIC DECORATION OF DNA ORIGAMI NANOSTRUCTURES WITH FUNCTIONALLY INTACT PROTEINS

Joschka Hellmeier[1,*], René Platzer[2], Vanessa Mühlgrabner[2], Magdalena C. Schneider[1], Elke Kurz[3], Gerhard J. Schütz[1], Johannes B. Huppa[2], Eva Sevcsik[1,*]

[1] Institute of Applied Physics, TU Wien, Vienna, Austria

[2] Center for Pathophysiology, Infectiology and Immunology, Institute for Hygiene and Applied Immunology, Medical University of Vienna, Vienna, Austria

[3] Kennedy Institute of Rheumatology, University of Oxford, Oxford, UK

* address correspondence to: eva.sevcsik@tuwien.ac.at, hellmeier@iap.tuwien.ac.at

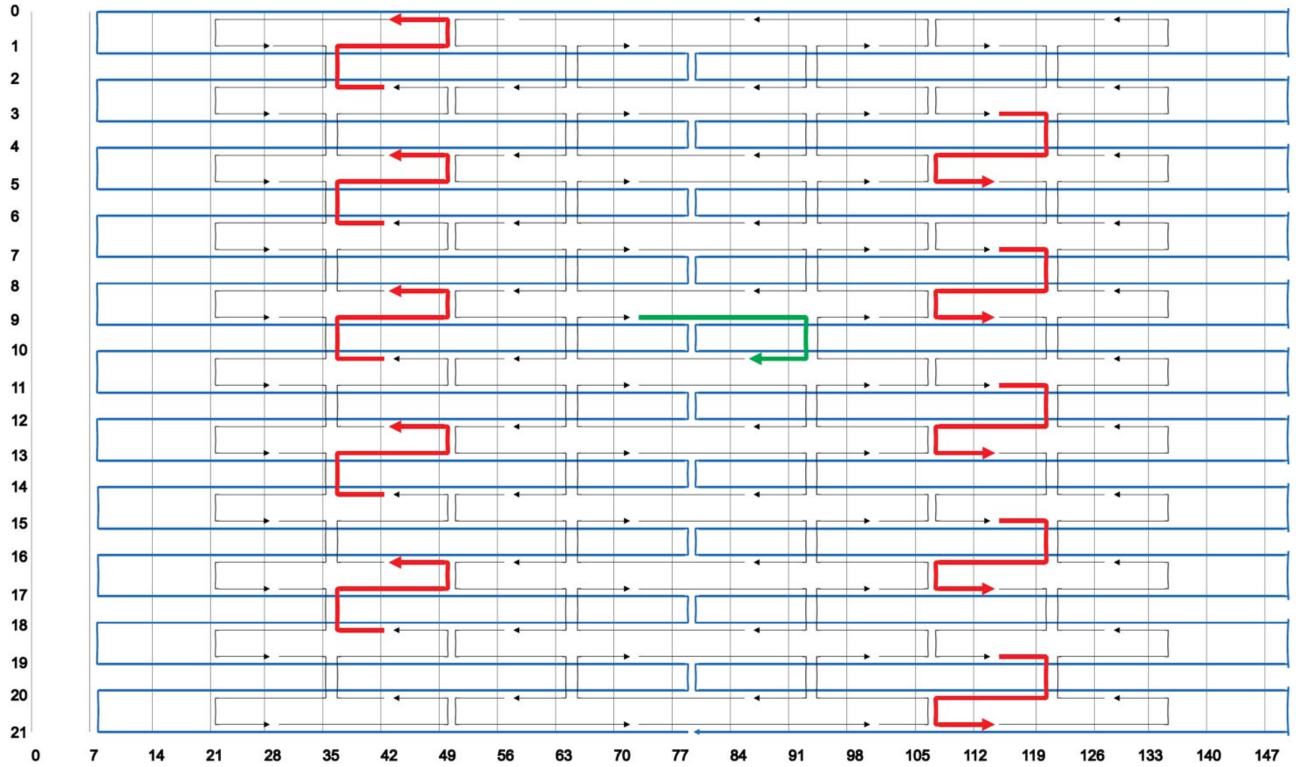

Fig. S1. DNA origami scaffold routing. The DNA origami rectangular tile (65x54 nm) was designed based on the M13mp18 scaffold[1] using caDNAno.[2] At the site chosen for ligand attachment, the staple strand was elongated at its 3'-end with 21 bases (green arrow). For attachment to the SLB *via* complementary cholesterol-oligonucleotides, staple strands were elongated at predefined positions at their 5'-end with 25 bases as indicated by the red arrows.

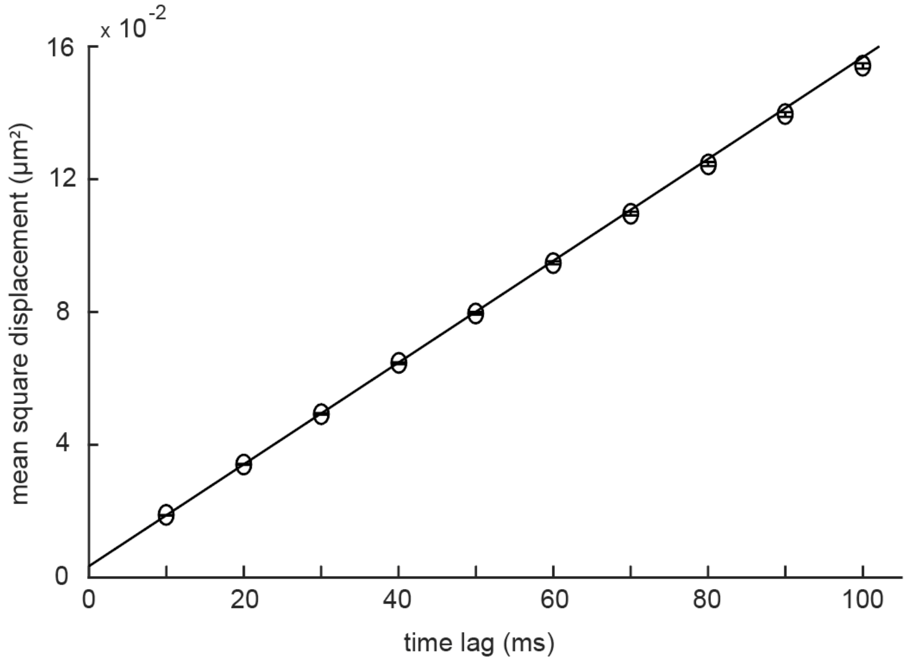

Fig. S2. Mobility of functionalized DNA origami structures on fluid-phase SLBs. Individual DNA origami structures were tracked on POPC SLBs at a frame rate of 100 Hz. Mean square displacements were determined and plotted as a function of time lags. Assuming free Brownian motion, the diffusion coefficient D was derived by fitting the first two data points with a linear fit. An exemplary plot for H57-dSAv is shown.

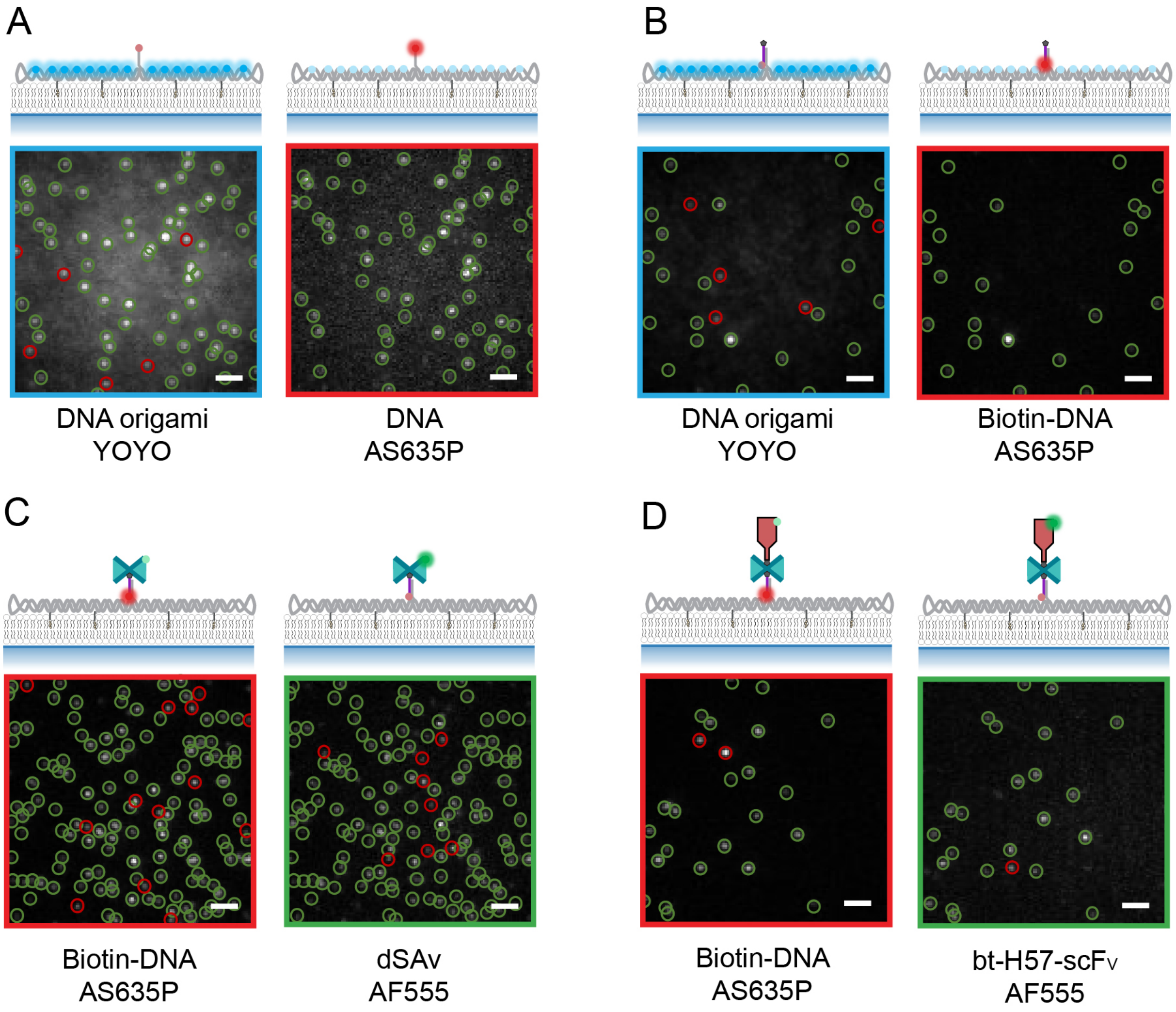

Fig. S3. Determining functionalization efficiencies for construct H57-dSAv. Single molecule two-color co-localization imaging was applied to determine the efficiency of each modification step. *(A)* First, we evaluated the availability of elongated staple strands (handles) for site-specific hybridization. For this purpose, we used fluorophore-conjugated handles (DNA-AS635P) and labeled the DNA origami structure with YOYO, a DNA-intercalating fluorophore. The percentage of co-localized signals in the blue (YOYO) and the red (DNA-AS635P) color channel yielded the handle incorporation efficiency. Representative TIRF images of DNA origami on an SLB are shown. Green open circles indicate signals detected in both color channels; red open circles indicate signals detected only in one channel. *(B)* Two-color colocalization of fluorescently labeled biotinylated oligo nucleotides (bt-DNA-AS635P) and YOYO yielded the efficiency of hybridization to the handle. *(C)* Binding of dSAv to the biotinylated handle was then determined *via* colocalization of fluorescently labeled biotinylated oligos (bt-DNA-AS635P) and dSAv (dSAv-AF555). *(D)* Finally, two-color colocalization of hybridized bt-DNA-AS635P and site-specifically biotinylated AF555-labeled H57-scFvs yielded the overall functionalization efficiency of DNA origami with the TCR-ligand. The functionalization efficiency was then assessed as described in the methods section. Scale bar, 2 µm.

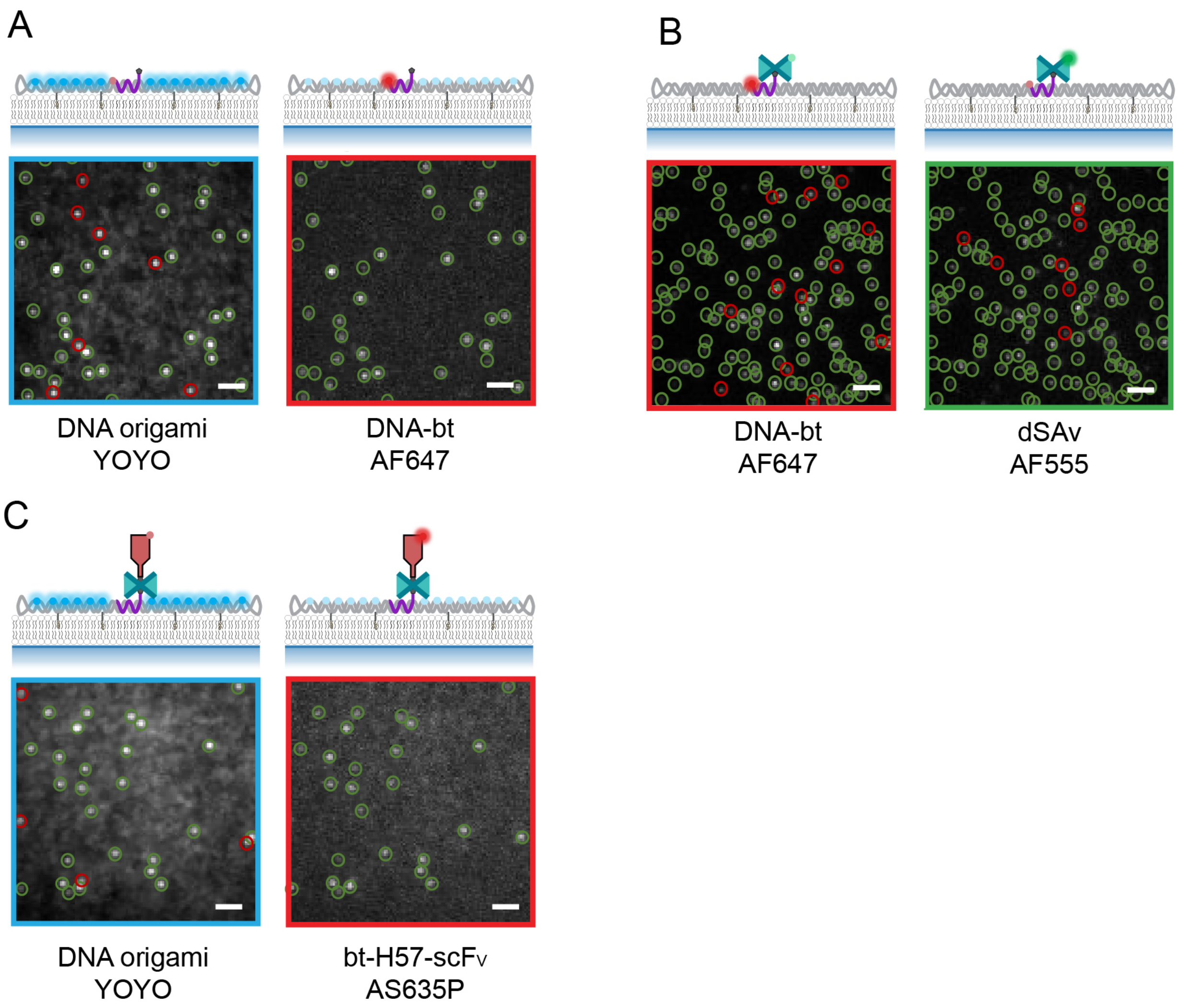

Fig. S4. Determining functionalization efficiencies for construct H57-dSAv-NL. *(A)* To determine the incorporation efficiency of the biotinylated handle, DNA origami structures featuring a fluorescently labeled, biotinylated handle (AF647-DNA-bt) were produced and unspecifically stained with the DNA-intercalating dye YOYO. Representative TIRF images of DNA origami on an SLB are shown. Green open circles indicate signals detected in both color channels; red open circles designate signals detected only in one channel. The percentage of co-localized signals in the blue (YOYO) and red (AF647-DNA-bt) color channel yielded the incorporation efficiency. (B) The binding efficiency of dSAv to the biotinylated handle was determined *via* two-color colocalization of fluorescently labeled dSAv (dSAv-AF555) and AF647-DNA-bt. (C) Two-color colocalization of AS635P-labeled H57-scFvs and YOYO-stained DNA origami structures yielded the overall functionalization efficiency of DNA origami with the TCR-ligand. Scale bar, 2 µm.

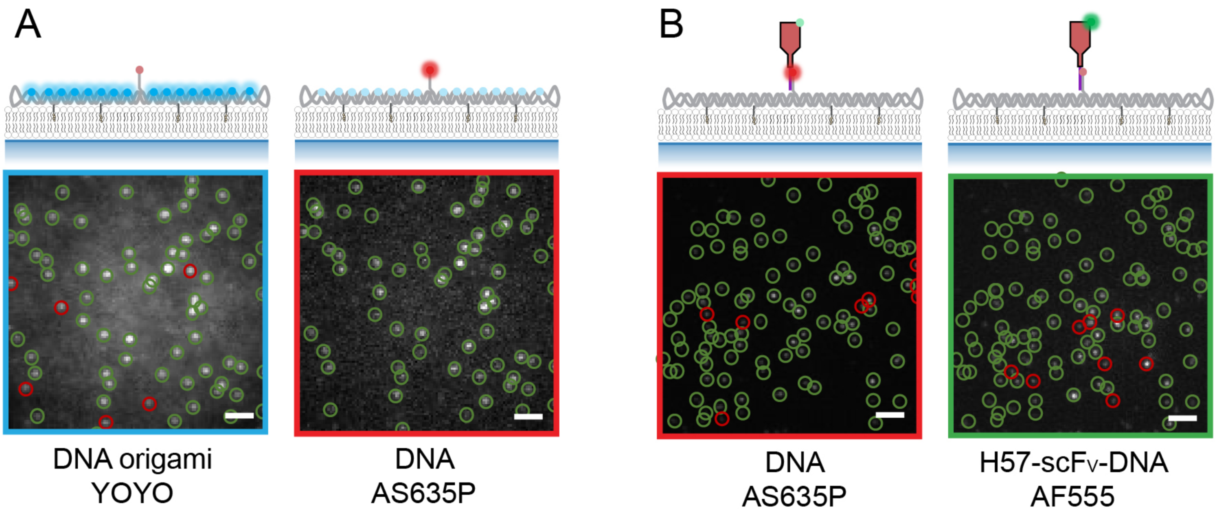

Fig. S5. Determining functionalization efficiencies for construct H57-DNA. *(A)* Handle availability was determined *via* two-color co-localization of DNA-AS635P and YOYO. Representative TIRF images of DNA origami on a SLB are shown. Green open circles indicate signals detected in both color channels; red open circles designate signals detected only in one channel. *(B)* Colocalization of AF555-labeled DNA-conjugated H57-scFvs (H57-DNA) and DNA-AS635P yielded the overall functionalization efficiency of DNA origami with ligand. Scale bar, 2 µm.

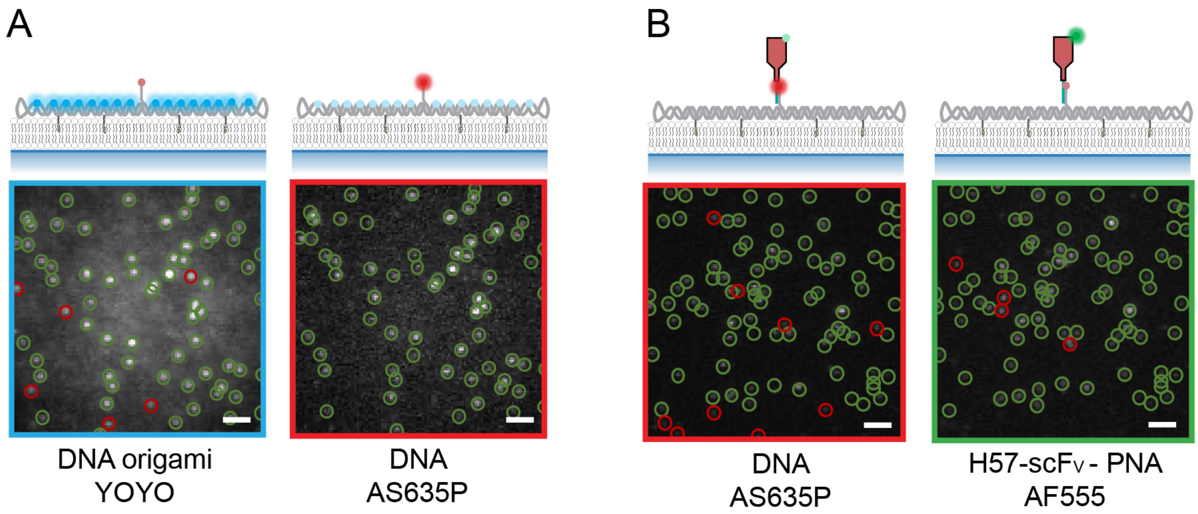

Fig. S6. Determining functionalization efficiencies for the construct H57-PNA. *(A)* Handle availability was determined *via* two-color co-localization of DNA-AS635P and YOYO. Exemplary TIRF images of DNA origami on a SLB are shown. Green open circles indicate signals detected in both color channels; red open circles designate signals detected only in one channel. *(B)* Colocalization of AF555-labeled PNA-conjugated H57-scFvs (H57-PNA) and DNA-AS635P yielded the overall functionalization efficiency of DNA origami with ligand. Scale bar, 2 µm.

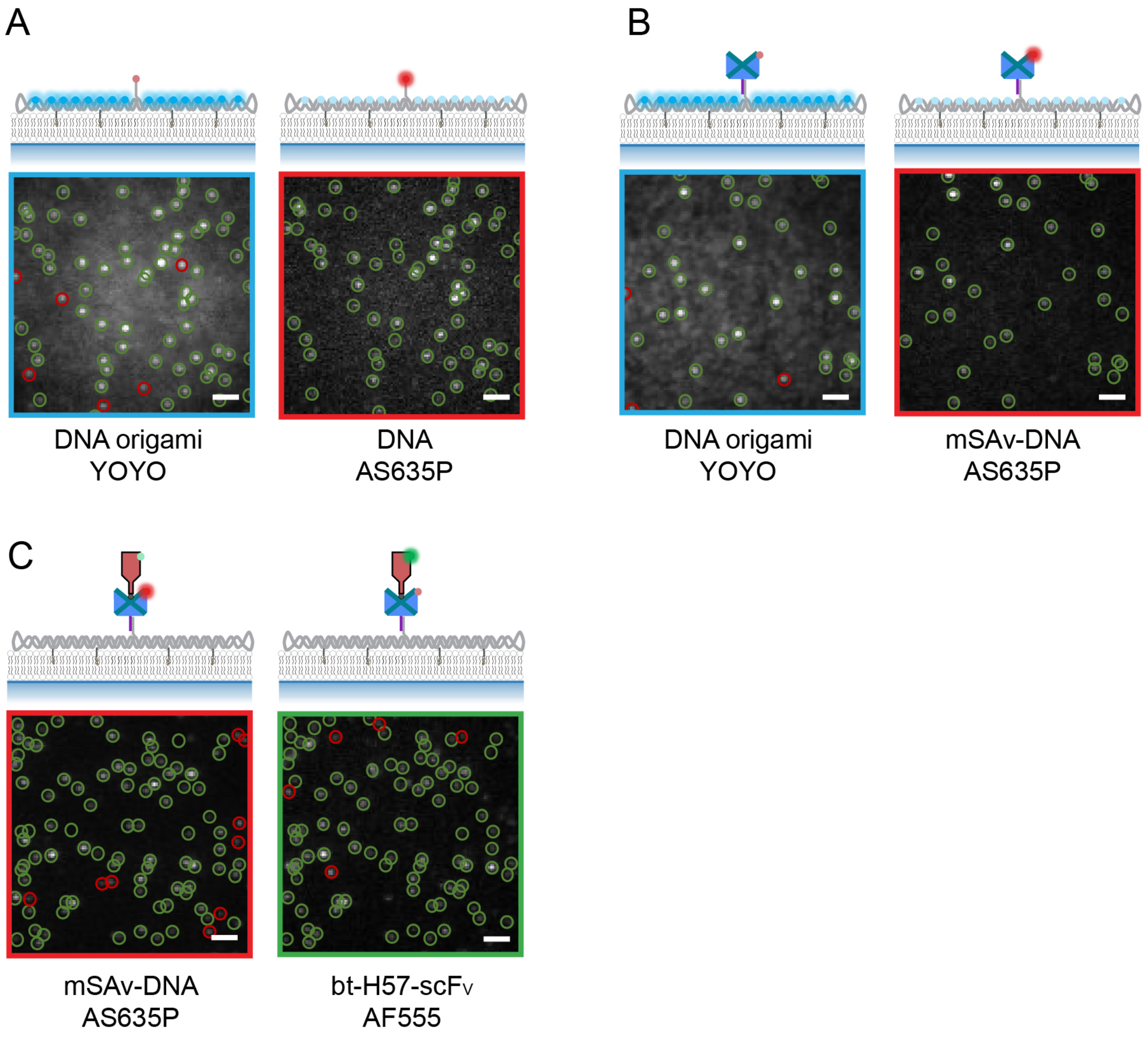

Fig. S7. Determining functionalization efficiencies for the construct H57-mSAv. *(A)* Handle availability was determined by two-color co-localization of DNA-AS635P and YOYO. Exemplary TIRF images of DNA origami on a SLB are shown. Green open circles indicate signals detected in both color channels; red open circles indicate signals detected only in one channel. *(B)* Colocalization of AS635P-labeled DNA-coupled mSAv (mSAv-DNA-AS635P) and YOYO yielded functionalization efficiency with mSAv. *(C)* Finally, two-color colocalization of mSAv-DNA-AS635P and AF555-labeled biotinylated H57-scFvs yielded the overall functionalization efficiency of DNA origami with ligand. Scale bar, 2 µm.

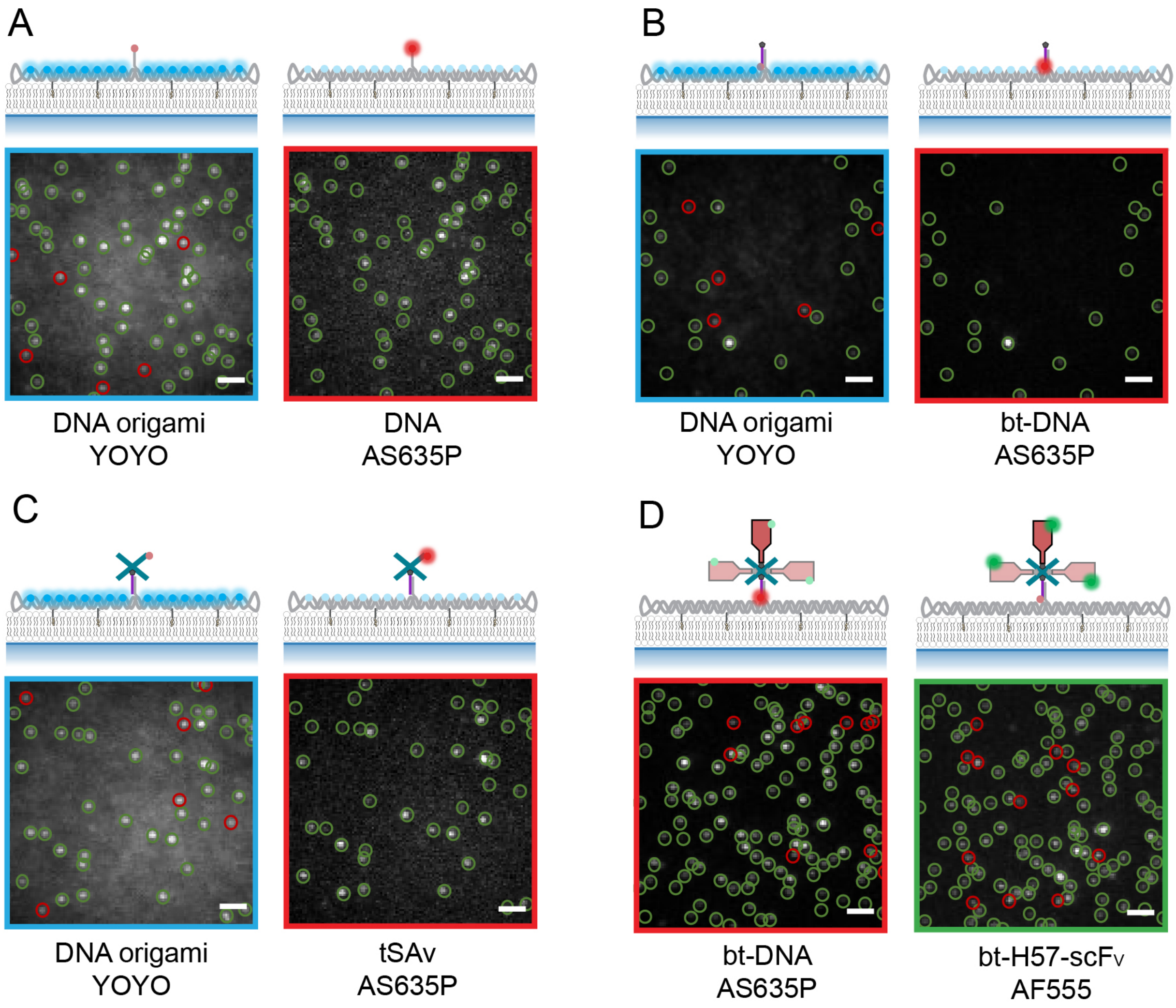

Fig. S8. Determining functionalization efficiencies for construct H57-tSAv. *(A)* Handle availability was determined by two-color co-localization of DNA-AS635P and YOYO. Exemplary TIRF images of DNA origami on a SLB are shown. Green open circles indicate signals detected in both color channels; red open circles indicate signals detected only in one channel. *(B)* Next, two-color colocalization of fluorescently labeled biotinylated oligos (bt-DNA-AS635P) with YOYO yielded the efficiency of hybridization of biotinylated oligo to the handle. *(C)* Colocalization of fluorescently labeled tSAv (tSAv-AS635P) and YOYO yielded functionalization efficiency with tSAv. *(D)* Finally, colocalization of hybridized biotin-DNA-AS635P and AF555-labeled H57-scFvs yielded the overall functionalization efficiency of DNA origami with ligand. Scale bar, 2 µm.

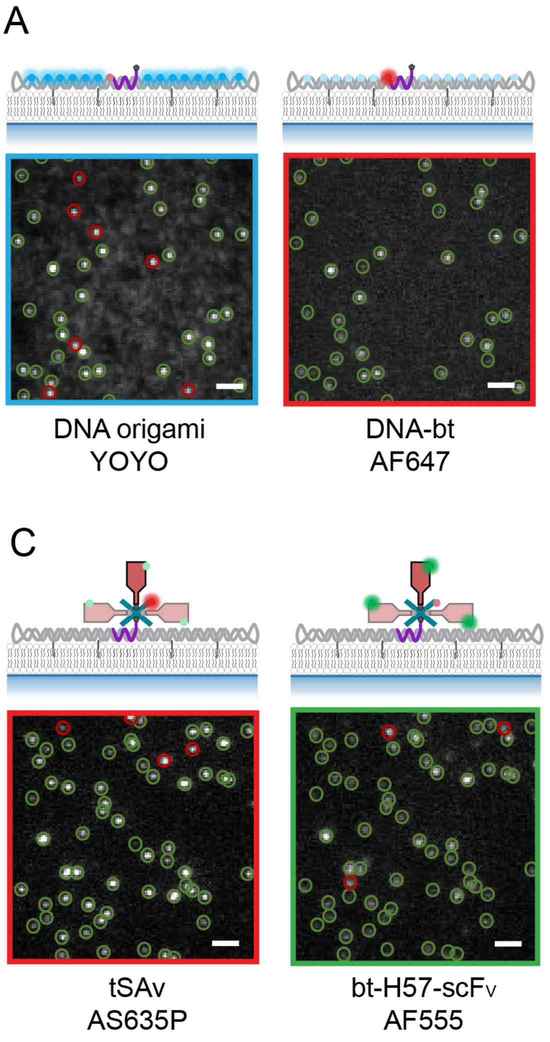

Fig. S9. Determining functionalization efficiencies for construct H57-tSAv-NL. *(A)* Incorporation of the biotinylated handle on DNA origami for functionalization was assessed *via* colocalization of a fluorescently labeled biotinylated oligo (AF647-DNA-bt) and YOYO. Exemplary TIRF images of DNA origami on a SLB are shown. Green open circles indicate signals detected in both color channels; red open circles indicate signals detected only in one channel. *(B)* The binding efficiency of tSAv to the biotinylated handle was determined *via* two-color colocalization of fluorescently labeled tSAv (tSAv-AS635P) and YOYO. *(C)* Last, two-color colocalization of tSAv-AS635P and AF555-labeled H57-scFvs yielded the overall functionalization efficiency of DNA origami with ligand. Scale bar, 2 µm.

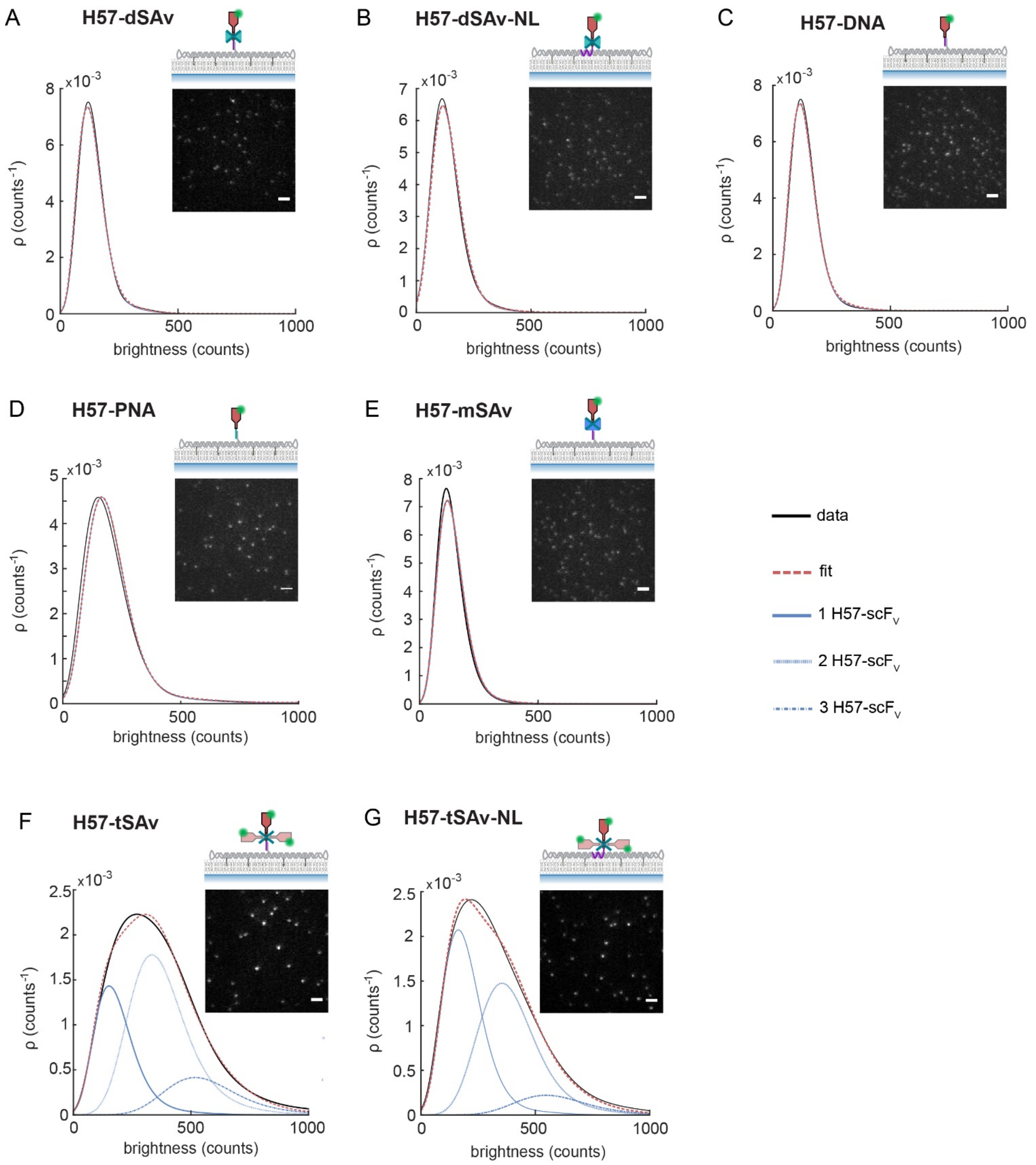

Fig. S10. Determining functionalization stoichiometries for H57-scF$_V$-functionalized DNA origami constructs. Exemplary TIRF images and corresponding brightness distributions ρ of DNA origami constructs functionalized with AF555-labeled H57-scF$_V$ on SLBs. The detected signals were fitted and deconvolved into monomer and multimer contributions[3] (see methods section). Scale bar, 2 µm.

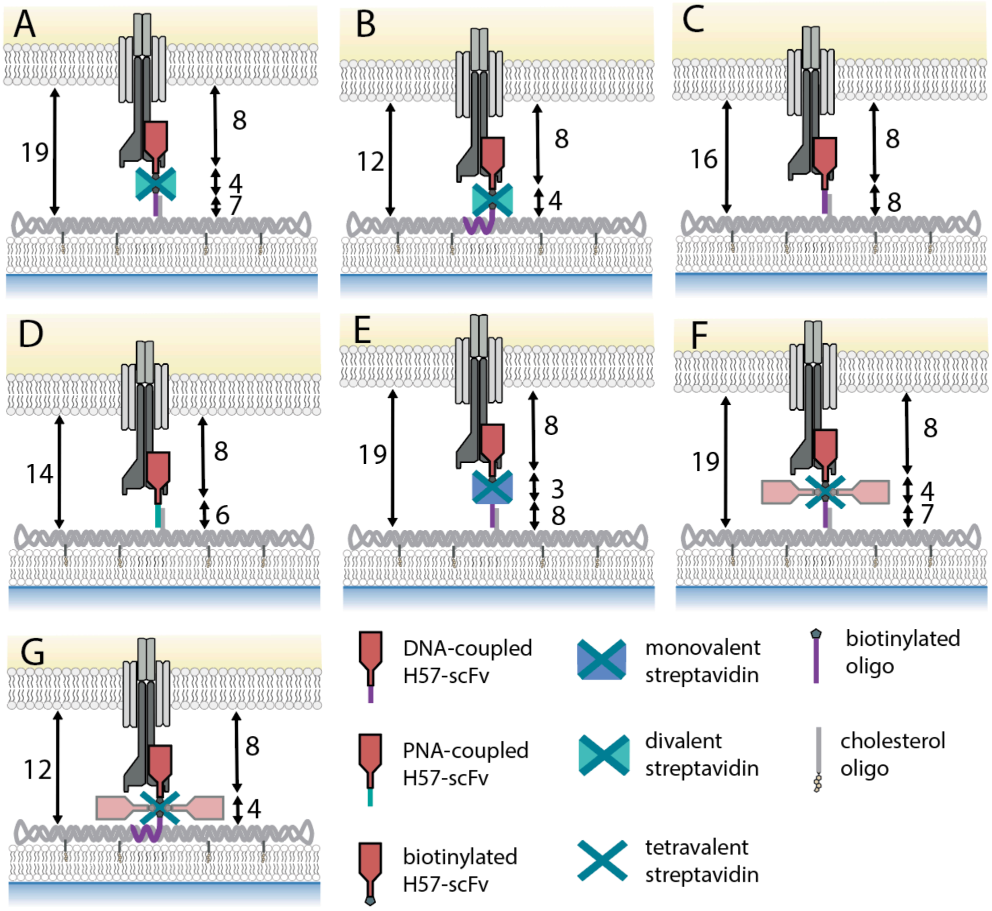

Fig. S11. Axial dimensions at the T-cell – SLB interface. Schematic sketches of TCR engagement for the different ligand-functionalized DNA origami structures: H57-dSAv *(A)*, H57-dSAv-NL *(B)*, H57-DNA *(C)*, H57-PNA *(D)*, H57-mSAv *(E)*, H57-tSAv *(F)*, H57-tSAv-NL *(G)*. Distances were estimated from the protein crystal structures[5–8] and are given in nm.

Note on length estimates

The two *trans* biotin binding sites in SAv are separated by ~3.5 nm;[8] hence the contribution to the total length of the construct was assumed with 4 nm *(A, F)*. For mSAv *(E)*, this contribution was estimated with 3 nm from the crystal structure.[7]

The DNA linker consists of 17 paired bases in constructs A, C, E, F; with the following variations:

A, F: (constructs H57-dSAv and H57-tSAv): 4 unpaired Ts on the handle, 17 paired bases, PEG4 linker and biotin. (~ 7 nm)

C, E: (constructs H57-DNA, H57-mSAv)): 4 unpaired Ts on the handle, 17 paired bases and 4 unpaired Ts on the hybridized DNA oligo, PEG4 linker (~ 8 nm)

D: The DNA/PNA linker consists of 4 unpaired Ts on the handle, 17 paired bases, 2 unpaired Ts and an O-linker (~ 6 nm).

B, G: (constructs H57-dSAv-NL and H57-tSAv-NL): The biotin is attached to the handle *via* 2 unpaired Ts and a PEG4 linker.

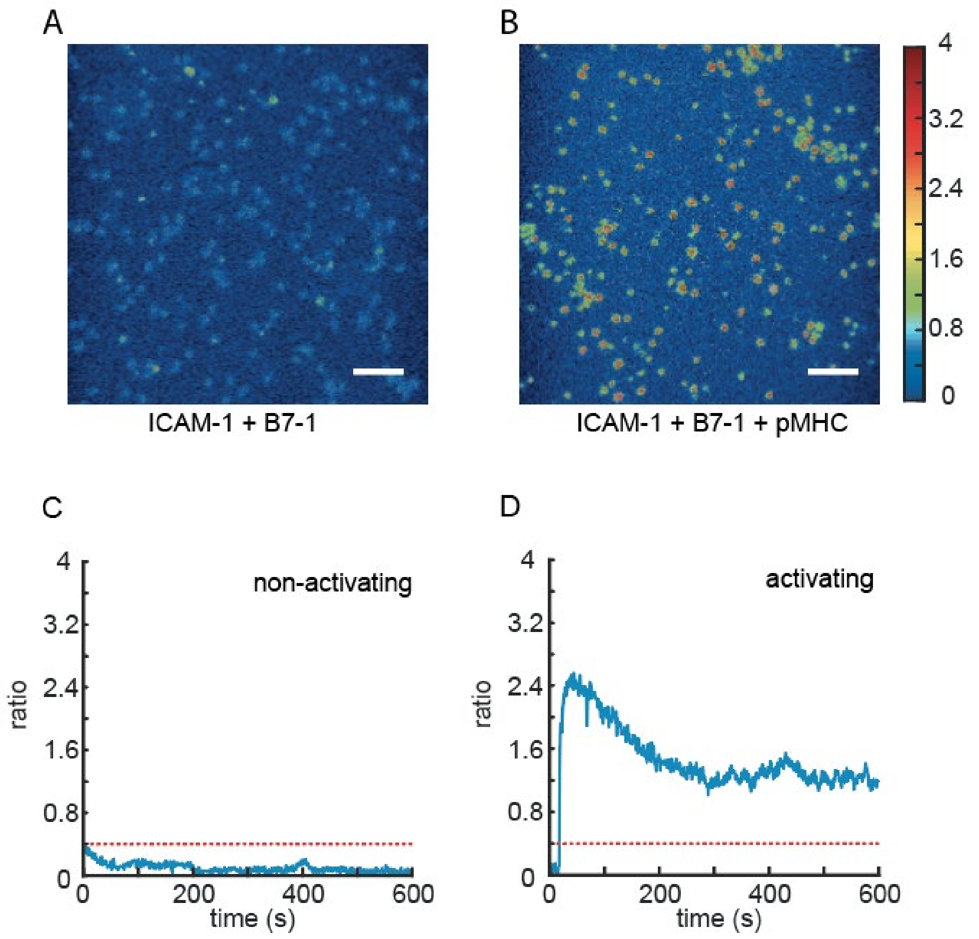

Fig. S12. Calcium imaging experiments to assess the T-cell activation state. T-cells were loaded with the ratiometric Ca^{2+}-sensitive dye Fura-2 AM, seeded onto SLBs and fluorescence emission was recorded at excitation wavelengths 340 nm and 380 nm over 10 min. Activation was tracked *via* a change of the intensity ratio (340/380nm). Exemplary ratio images recorded at activating (ICAM-1 100 μm^{-2}, B7-1 100 μm^{-2}, pMHC 150 μm^{-2}, *(A))* and non-activating (ICAM-1 100 μm^{-2}, B7-1 100 μm^{-2}, *(B))* conditions at 37°C are shown 5 min after cell seeding. Scale bar, 4 µm. *(C,D)* For each cell, the intensity ratio 340/380nm was determined and plotted over time. Exemplary calcium traces for a T-cell under non-activating *(C)* and activating *(D)* conditions are shown. The threshold ratio for counting a cell as “activated” was set to 0.4 for all experiments, indicated by a red dashed line.

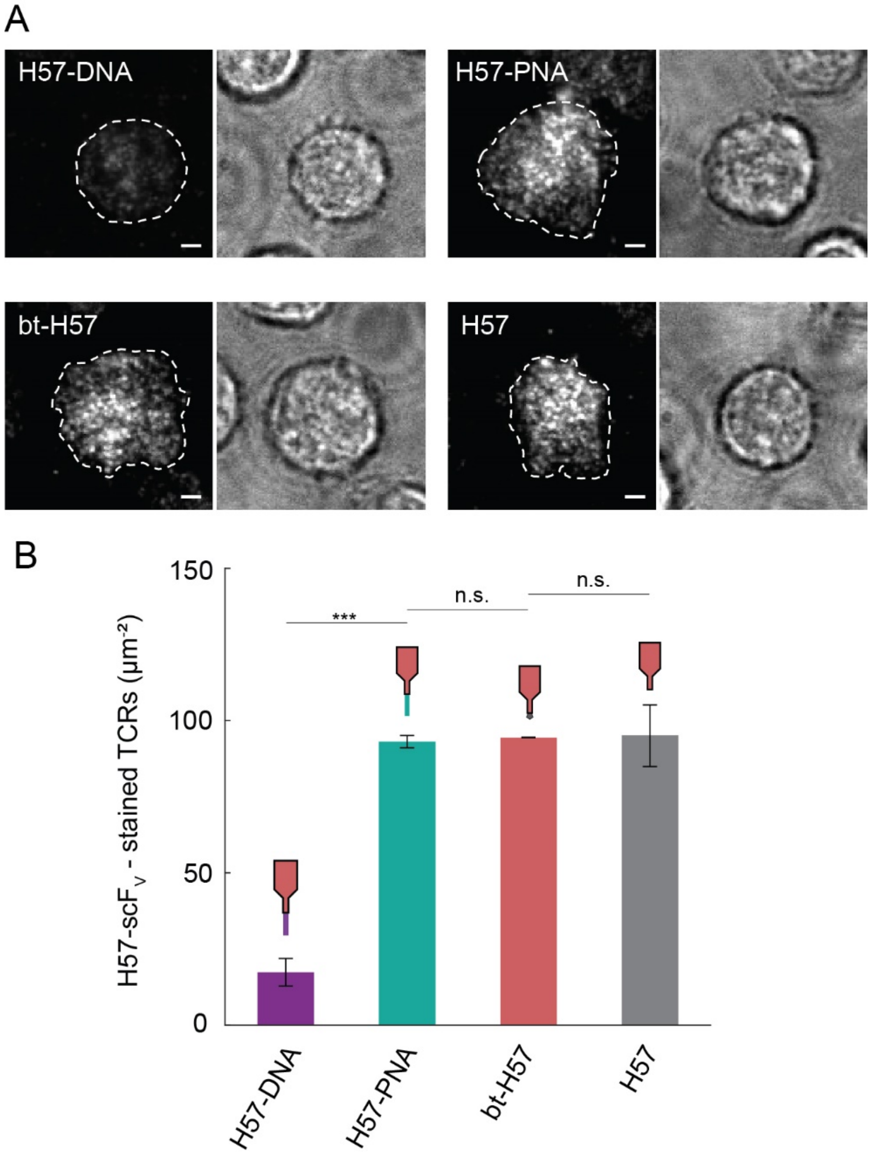

Fig. S13. Determination of the TCR labeling efficiency of the different H57-scF$_V$ variants. *(A)* TCRs on T-cells were stained with different AF555-conjugated H57-scF$_V$ variants (H57-DNA, H57-PNA, bt-H57, H57) and allowed to adhere to SLBs presenting 100 molecules per µm^2 ICAM-1 for imaging in TIRF microscopy mode. T-cells were labeled under saturating conditions for AF555-conjugated H57-scF$_V$ (20 µg ml^{-1}).[4] The cell outline is indicated by a dashed white contour line. Images were recorded 5 -10 min after cell seeding. Scale bar, 2 µm. (B) Surface densities of labeled TCRs were quantified ($n \geq 27$ cells). Data are the mean of two independent experiments and two different mice (± s.e.m.).

Table S1. Diffusion coefficients of constructs on SLBs. Single-molecule trajectories of DNA origami structures functionalized with H57-scF$_V$ were recorded on SLBs, pooled and diffusion coefficients were determined by mean square displacement analysis. Diffusion coefficients are given as mean ± s.e.m. Data are from at least two independent experiments.

Construct	D (µm^2/s)	trajectories (n)
**H57-dSAv**	0.380 ± 0.013	8,203
**H57-dSAv-NL**	0.381 ± 0.015	9,857
**H57-DNA**	0.383 ± 0.012	7,601
**H57-PNA**	0.421 ± 0.003	3,797
**H57-mSAv**	0.385 ± 0.012	8,669
**H57-tSAv**	0.355 ± 0.015	11,108
**H57-tSAv-NL**	0.399 ± 0.001	5,699

Table S2. Fluorophore pairs used for two-color colocalization microscopy.

Construct	Fluorophore ID	Handle incorporation	bt-DNA hybridization	SAv attachment	POI attachment
**1: H57-dSAv**	DYE 1	DNA origami (YOYO)	DNA origami (YOYO)	bt-DNA (AS635P)	bt-DNA (AS635P)
	DYE 2	DNA (AS635P)	bt-DNA (AS635P)	dSAv (AF555)	bt-H57 (AF555)
**2: H57-dSAv-NL**	DYE 1	DNA origami (YOYO)	---	DNA (AS635P)	DNA origami (YOYO)
	DYE 2	DNA-bt (AF647)	---	dSAv (AF555)	bt-H57 (AS635P)
**3: H57-DNA**	DYE 1	DNA origami (YOYO)	---	---	DNA (AS635P)
	DYE 2	DNA (AS635P)	---	---	H57-DNA (AF555)
**4: H57-PNA**	DYE 1	DNA origami (YOYO)	---	---	DNA (AS635P)
	DYE 2	DNA (AS635P)	---	---	H57-PNA (AF555)
**5: H57-mSAv**	DYE 1	DNA origami (YOYO)	---	DNA origami (YOYO)	mSAv-DNA (AS635P)
	DYE 2	DNA (AS635P)	---	mSAv-DNA (AS635P)	bt-H57 (AF555)
**6: H57-tSAv**	DYE 1	DNA origami (YOYO)	DNA origami (YOYO)	DNA origami (YOYO)	bt-DNA (AS635P)
	DYE 2	DNA (AS635P)	bt-DNA (AS635P)	tSAv (AS635P)	bt-H57 (AF555)
**7: H57-tSAv-NL**	DYE 1	DNA origami (YOYO)	---	DNA origami (YOYO)	tSAv (AS635P)
	DYE 2	DNA-bt (AF647)	---	tSAv (AS635P)	bt-H57 (AF555)

Table S3. Optimization of functionalization conditions for construct H57-dSAv. For each step, the yield of functionalized DNA origami construct was determined for different conditions (incubation times and molar ratios) *via* two-color colocalization microscopy as sketched in SI Fig. S3. Optimal conditions for each step are marked in red; these were then used as the basis for subsequent steps. Cumulative yields are shown. Data are the mean (± s.e.m.) of at least two independent experiments.

Handle incorporation						
Molar ratio DNA origami : handle oligo						
1 : 1	1 : 2	1 : 5	1 : 10	1 : 30	1 : 50	1 : 100
19.1 ± 0.9	32.8 ± 0.8	46.6 ± 0.6	84.4 ± 2.1	83.4 ± 2.7	86.4 ± 0.3	86.4 ± 0.0

bt-DNA hybridization					
Molar ratio DNA origami : bt-DNA					
1 : 10	1 : 20	1 : 50	1 : 100	1 : 300	1 : 1000
26.9 ± 0.3	39.1 ± 2.0	86.4 ± 0.5	85.4 ± 0.3	85.4 ± 0.2	85.9 ± 0.0

SAv attachment				
Molar ratio DNA origami : dSAv				
1 : 1	1 : 5	1 : 10	1 : 100	Incubation time [min]
33.1 ± 2.4	34.1 ± 0.6	32.6 ± 1.5	67.1 ± 0.6	10
34.3 ± 1.7	61.0 ± 0.8	68.7 ± 0.4	67.9 ± 1.2	30
34.2 ± 1.0	61.2 ± 0.7	68.1 ± 1.2	67.6 ± 1.5	60
33.7 ± 0.5	65.0 ± 1.4	68.4 ± 0.9	68.5 ± 0.5	300

POI attachment				
Molar ratio DNA origami : bt-H57-scF$_V$				
1 : 1	1 : 5	1 : 10	1 : 100	Incubation time [min]
34.7 ± 2.2	59.8 ± 0.9	62.0 ± 0.4	66.9 ± 1.5	10
32.0 ± 1.2	59.1 ± 1.5	62.2 ± 1.3	67.5 ± 0.5	30
31.1 ± 1.4	60.1 ± 0.8	67.4 ± 1.0	68.1 ± 0.7	60
34.6 ± 1.0	66.6 ± 0.9	67.3 ± 1.0	67.5 ± 0.5	300

Table S4. Optimization of functionalization conditions for construct H57-dSAv-NL. For each step, the yield of functionalized DNA origami construct was determined for different conditions (molar ratios, incubation times) *via* two-color colocalization microscopy as sketched in SI Fig. S4. Cumulative yields are shown. Optimal conditions for each step are marked in red. Data are the mean (± sem) of at least two independent experiments.

**Handle incorporation**					
**Molar ratio DNA origami : biotinylated handle oligo**					
**1 : 1**	**1 : 2**	**1 : 5**	**1 : 10**	**1 : 30**	**1 : 100**
**21.2 ± 1.2**	**53.8 ± 7.0**	**59.0 ± 7.0**	**84.0 ± 2.7**	**79.9 ± 6.2**	**79.4 ± 0.5**

**SAv attachment**				
**Molar ratio DNA origami : dSAv**				
**1 : 1**	**1 : 5**	**1 : 10**	**1 : 100**	**Incubation time [min]**
**43.2 ± 2.7**	**44.6 ± 6.6**	**64.7 ± 2.8**	**66.5 ± 3.8**	**10**
**46.8 ± 4.6**	**55.4 ± 8.6**	**71.7 ± 3.3**	**67.8 ± 2.6**	**30**
**44.2 ± 1.9**	**67.4 ± 3.6**	**72.0 ± 2.6**	**68.0 ± 2.5**	**60**
**46.1 ± 8.2**	**66.3 ± 3.0**	**69.1 ± 4.2**	**70.1 ± 3.1**	**300**

**POI attachment**				
**Molar ratio DNA origami : bt-H57-scF$_V$**				
**1 : 1**	**1 : 5**	**1 : 10**	**1 : 100**	**Incubation time [min]**
**58.4 ± 4.8**	**63.5 ± 4.9**	**66.2 ± 4.5**	**68.5 ± 4.5**	**10**
**59.5 ± 5.7**	**66.1 ± 3.2**	**67.0 ± 5.0**	**69.6 ± 3.7**	**30**
**64.0 ± 2.4**	**66.4 ± 4.0**	**71.2 ± 2.6**	**69.8 ± 3.2**	**60**
**64.3 ± 5.3**	**67.4 ± 4.5**	**72.4 ± 0.3**	**73.2 ± 2.5**	**300**

Table S5. Optimization of functionalization conditions for construct H57-DNA. For each step, the yield of functionalized DNA origami construct was determined for different conditions (molar ratios, incubation times) *via* two-color colocalization microscopy as sketched in SI Fig. S5. Cumulative yields are shown. Optimal conditions for each step are marked in red. Data are the mean (± sem) of at least two independent experiments.

**Handle incorporation**						
**Molar ratio DNA origami : handle oligo**						
**1 : 1**	**1 : 2**	**1 : 5**	**1 : 10**	**1 : 30**	**1 : 50**	**1 : 100**
**19.1 ± 0.9**	**32.8 ± 0.8**	**46.6 ± 0.6**	**84.4 ± 2.1**	**83.4 ± 2.7**	**86.4 ± 0.3**	**86.4 ± 0.0**

**POI attachment**				
**Molar ratio DNA origami : H57-scF$_V$-DNA**				
**1 : 1**	**1 : 5**	**1 : 10**	**1 : 100**	**Incubation time [min]**
**32.6 ± 2.6**	**62.8 ± 2.9**	**65.7 ± 1.9**	**67.1 ± 3.0**	**10**
**34.9 ± 4.3**	**63.7 ± 2.5**	**66.9 ± 2.4**	**67.6 ± 1.9**	**30**
**36.1 ± 3.3**	**65.4 ± 4.1**	**67.9 ± 2.4**	**67.6 ± 2.1**	**60**
**58.5 ± 1.7**	**66.3 ± 3.6**	**67.7 ± 1.8**	**67.8 ± 2.6**	**300**

Table S6. Determination of functionalization yields after each step for construct H57-PNA. For each step, the yield of functionalized DNA origami construct was determined for different conditions (incubation times and molar ratios) *via* two-color colocalization microscopy as sketched in SI Fig. S6. Cumulative yields are shown. Optimal conditions for each step are marked in red. Data are the mean (± s.e.m.) of at least two independent experiments.

**Handle incorporation**						
**Molar ratio DNA origami : handle oligo**						
**1 : 1**	**1 : 2**	**1 : 5**	**1 : 10**	**1 : 30**	**1 : 50**	**1 : 100**
**19.1 ± 0.9**	**32.8 ± 0.8**	**46.6 ± 0.6**	**84.4 ± 2.1**	**83.4 ± 2.7**	**86.4 ± 0.3**	**86.4 ± 0.0**

**POI attachment**				
**Molar ratio DNA origami : H57-scF$_V$-PNA**				
**1 : 1**	**1 : 3**	**1 : 10**	**1 : 100**	**Incubation time [min]**
**24.7 ± 1.9**	**28.0 ± 1.5**	**47.9 ± 3.2**	**48.9 ± 3.2**	**10**
**50.2 ± 6.0**	**71.5 ± 2.5**	**71.7 ± 2.3**	**73.1 ± 2.4**	**30**
**52.0 ± 4.5**	**74.2 ± 2.2**	**72.9 ± 2.5**	**73.0 ± 3.0**	**60**
**51.4 ± 5.0**	**73.6 ± 2.0**	**72.9 ± 2.5**	**72.9 ± 2.7**	**300**

Table S7. Determination of functionalization yields after each step for construct H57-mSAv. For each step, the yield of functionalized DNA origami construct was determined for different conditions (incubation times and molar ratios) *via* two-color colocalization microscopy as sketched in SI Fig. S7. Cumulative yields are shown. Optimal conditions for each step are marked in red. Data are the mean (± s.e.m.) of at least two independent experiments.

.

Handle incorporation						
Molar ratio DNA origami : handle oligo						
1 : 1	1 : 2	1 : 5	1 : 10	1 : 30	1 : 50	1 : 100
19.1 ± 0.9	32.8 ± 0.8	46.6 ± 0.6	84.4 ± 2.1	83.4 ± 2.7	86.4 ± 0.3	86.4 ± 0.0

SAv attachment			
Molar ratio DNA origami : mSAv-DNA			
1 : 3	1 : 10	1 : 100	Incubation time [min]
68.8 ± 2.7	74.0 ± 2.5	76.4 ± 3.1	10
75.4 ± 0.4	75.7 ± 1.5	78.1 ± 0.6	30
81.0 ± 2.4	78.5 ± 2.7	78.2 ± 5.8	60
78.0 ± 0.6	80.0 ± 1.9	81.1 ± 3.0	300

POI attachment				
Molar ratio DNA origami : bt-H57-scF$_V$				
1 : 1	1 : 5	1 : 10	1 : 100	Incubation time [min]
47.4 ± 3.0	50.0 ± 4.8	56.4 ± 6.3	62.1 ± 2.6	10
49.5 ± 1.5	58.3 ± 5.0	63.2 ± 2.4	65.5 ± 3.0	30
50.7 ± 1.6	63.1 ± 1.9	66.6 ± 3.2	66.3 ± 2.9	60
53.7 ± 1.7	65.5 ± 3.2	66.5 ± 3.1	67.6 ± 3.8	300

Table S8. Determination of functionalization yields after each step for construct H57-tSAv. For each step, the yield of functionalized DNA origami construct was determined for different conditions (incubation times and molar ratios) *via* two-color colocalization microscopy as sketched in SI Fig. S8. Cumulative yields are shown. Optimal conditions for each step are marked in red. Data are the mean (± s.e.m.) of at least two independent experiments.

**Handle incorporation**						
**Molar ratio DNA origami : handle oligo**						
**1 : 1**	**1 : 2**	**1 : 5**	**1 : 10**	**1 : 30**	**1 : 50**	**1 : 100**
**19.1 ± 0.9**	**32.8 ± 0.8**	**46.6 ± 0.6**	**84.4 ± 2.1**	**83.4 ± 2.7**	**86.4 ± 0.3**	**86.4 ± 0.0**

**bt-DNA hybridization**					
**Molar ratio DNA origami : bt-DNA**					
**1 : 10**	**1 : 20**	**1 : 50**	**1 : 100**	**1 : 300**	**1 : 1000**
**26.9 ± 0.3**	**39.1 ± 2.0**	**86.4 ± 0.5**	**85.4 ± 0.3**	**85.4 ± 0.2**	**85.9 ± 0.0**

**SAv attachment**				
**Molar ratio DNA origami : tSAv**				
**1 : 1**	**1 : 5**	**1 : 10**	**1 : 100**	**Incubation time [min]**
**76.6 ± 4.8**	**77.9 ± 5.2**	**77.5 ± 5.7**	**79.0 ± 5.6**	**10**
**77.5 ± 3.6**	**79.7 ± 4.8**	**79.4 ± 3.2**	**79.4 ± 3.6**	**30**
**76.8 ± 4.3**	**78.3 ± 6.0**	**79.6 ± 4.0**	**79.3 ± 4.0**	**60**
**77.7 ± 4.9**	**78.8 ± 6.1**	**79.6 ± 4.0**	**82.1 ± 2.6**	**300**

**POI attachment**	
**Molar ratio DNA origami : bt-H57-scF$_V$**	
**1 : 10**	**Incubation time [min]**
**69.1 ± 2.3**	**10**
**70.5 ± 1.1**	**30**
**70.1 ± 1.3**	**60**
**68.1 ± 3.0**	**300**

Table S9. Determination of functionalization yields after each step for construct H57-tSAv-NL. For each step, the yield of functionalized DNA origami construct was determined for different conditions (incubation times and molar ratios) *via* two-color colocalization microscopy as sketched in SI Fig. S9. Cumulative yields are shown. Optimal conditions for each step are marked in red. Data are the mean (± s.e.m.) of at least two independent experiments.

**Handle incorporation**					
**Molar ratio DNA origami : biotinylated handle oligo**					
**1 : 1**	**1 : 2**	**1 : 5**	**1 : 10**	**1 : 30**	**1 : 100**
**21.2 ± 1.2**	**53.8 ± 7.0**	**59.0 ± 7.0**	**84.0 ± 2.7**	**79.9 ± 6.2**	**79.4 ± 0.5**

**SAv attachment**				
**Molar ratio DNA origami : tSAv**				
**1 : 1**	**1 : 5**	**1 : 10**	**1 : 100**	**Incubation time [min]**
**65.2 ± 6.5**	**78.8 ± 0.8**	**79.5 ± 1.4**	**81.2 ± 1.7**	**10**
**67.9 ± 10.5**	**79.5 ± 1.5**	**83.8 ± 1.3**	**81.3 ± 1.7**	**30**
**78.0 ± 2.4**	**80.6 ± 2.3**	**82.7 ± 0.3**	**81.5 ± 1.8**	**60**
**81.0 ± 1.4**	**81.6 ± 1.1**	**82.1 ± 2.4**	**83.9 ± 0.2**	**300**

**POI attachment**	
**Molar ratio DNA origami : bt-H57-scF$_V$**	
**1 : 10**	**Incubation time [min]**
**71.0 ± 1.3**	**10**
**71.2 ± 1.2**	**30**
**72.1 ± 1.9**	**60**
**71.6 ± 1.8**	**300**

Table S10. Functionalization efficiencies for the different constructs. The degree of functionalization (%, ± s.e.m.) (with one or more ligands) was determined *via* two-color colocalization microscopy (see Figures SI 3-9). Cumulative yields are shown in *(A).* To allow for direct comparison of the efficiencies of individual functionalization steps we provide this information in (B). Note that for *(B)* some step efficiencies could not be determined directly but had to be calculated (indicated with #).

A	H57-dSAv	H57-dSAv-NL	H57-DNA	H57-PNA	H57-mSAv	H57-tSAv	H57-tSAv-NL
**handle incorporation**	84.4 ± 2.1	84.0 ± 2.7	84.4 ± 2.1	84.4 ± 2.1	84.4 ± 2.1	84.4 ± 2.1	84.0 ± 2.7
**bt-DNA hybridization**	86.4 ± 0.5	-	-	-	-	86.4 ± 0.5	-
**SAv attachment**	68.7 ± 0.1	72.0 ± 0.2	-	-	81.0 ± 2.4*	79.4 ± 3.2	83.8 ± 1.3
**H57-scFv attachment**	67.4 ± 0.7	71.2 ± 2.6	67.9 ± 0.7*	74.2 ± 0.3*	66.6 ± 1.2	70.1 ± 0.9	72.1 ± 0.7
B	**H57-dSAv**	**H57-dSAv-NL**	**H57-DNA**	**H57-PNA**	**H57-mSAv**	**H57-tSAv**	**H57-tSAv-NL**
**handle incorporation**	84.0 ± 2.7	84.0 ± 2.7	84.4 ± 2.1	84.4 ± 2.1	84.4 ± 2.1	84.4 ± 2.1	84.0 ± 2.7
**bt-DNA hybridization**	102.4 ± 3.1#	-	-	-	-	102.4 ± 3.1#	-
**SAv attachment**	85.7 ± 3.0	85.7 ± 3.0	-	-	96.0 ± 5.2*#	91.9 ± 6.5#	99.8 ± 4.8#
**H57-scFv attachment**	98.9 ± 3.9#	98.9 ± 3.9#	80.5 ± 2.8*	87.9 ± 2.5*	82.2 ± 3.9	88.3 ± 7.4#	86.0 ± 2.2

*indicates attachment *via* hybridization

Table S11. Number of H57-scF$_V$ molecules per DNA origami construct. The number of H57-scF$_V$ molecules per functionalized DNA origami construct was determined by comparing the signal brightness of the construct to the brightness of a single AF555-labeled H57-scF$_V$ as described in the methods section. Data are the mean of at least two independent experiments (± s.e.m.).

Construct	1 H57-scF$_V$	2 H57-scF$_V$	3 H57-scF$_V$	signals (n)
**H57-dSAv**	98.6 ± 0.8	0.5 ± 0.3	0.6 ± 0.5	1,352 ± 147
**H57-dSAv-NL**	99.9 ± 0.3	0.1 ± 0.1	0.6 ± 0.2	1,369 ± 107
**H57-DNA**	97.5 ± 1.1	2.2 ± 1.1	0.3 ± 0.2	1,273 ± 69
**H57-PNA**	99.5 ± 0.2	0.0 ± 0.0	0.5 ± 0.2	2,276 ± 484
**H57-mSAv**	98.6 ± 0.8	1.1 ± 0.7	0.3 ± 0.1	1,359 ± 63
**H57-tSAv**	30.6 ± 1.8	52.8 ± 3.2	16.7 ± 5.1	5,984 ± 1,627
**H57-tSAv-NL**	48.1 ± 3.2	47.1 ± 0.7	4.8 ± 3.9	3,096 ± 565

Table S12. Fitting parameters of fits to dose-response curves. Dose-response curves of T-cell activation by *via* the different ligand-decorated DNA origami constructs were fitted with Eq. 19 to extract the activation threshold T_A, the maximum response A_{max} and the Hill coefficient n. The 95% confidence intervals are indicated. The mean number of cells per region (± s.e.m.) and the number of animals used to generate dose-response curves are shown.

Construct	n	n low	n high	A max (%)	A max, low	A max, high	T_A (µm^{-2})	$T_{A,}$ low	$T_{A,}$ high	# cells	# animals
**H57-dSAv**	2.44	1.66	3.23	91.81	84.50	99.13	3.91	3.29	4.64	163 ± 35	3
**H57-dSAv-NL**	3.06	1.83	4.29	100.14	91.55	108.73	3.27	2.78	3.85	199 ± 55	2
**H57-DNA**	4.38	2.49	6.26	91.01	81.41	100.62	9.68	8.66	10.83	176 ± 68	2
**H57-PNA**	1.73	0.94	2.52	103.83	94.52	113.15	2.75	2.20	3.43	206 ± 65	2
**H57-mSAv**	3.35	0.99	5.71	92.55	84.01	101.09	3.15	2.58	3.86	203 ± 53	2
**H57-tSAv**	1.23	0.33	2.12	105.74	89.16	122.31	0.81	0.45	1.49	289 ± 81	2
**H57-tSAv-NL**	1.42	0.72	2.12	101.65	90.28	113.02	0.83	0.45	1.54	214 ± 62	2

Table S13. List of staple strands

Designation	Sequence
10[63]-BLK	CAGCTTTCGGGCGCATCGTAACCGATCGGCCT
11[112]-BLK	AGCGCGAACAATCATAAGGGAACCCGGTGTAC
12[95]-BLK	ACACTCATAGGGGACGACGACAGTTGCATCTG
13[80]-BLK	CGCCAGCTAAAGACAGCATCGGAAGTCACCCT
14[63]-BLK	GCGATTAAGTACCGAGCTCGAATTAATTGTTA
15[112]-BLK	TTTCTTAACCGCTTTTGCGGGATCCGAGGGTA
16[95]-BLK	GTATCGGTGCTGTTTCCTGTGTGACGTAATCA
19[112]-BLK	TAGCAAGCCGATCTAAAGTTTTGTGTATGGGA
2[63]-BLK	TATTTTCATTAAGCAATAAAGCCTACATTATG
5[80]-BLK	CGGAGACATTCATCAGTTGAGATTATTACAGG
6[63]-BLK	TCAACCGTATCGATGAACGGTAATGGTTGATA
7[112]-BLK	TAATTTCACTAACGGAACAACATTTAGGAATA
8[95]-BLK	AGAACGAGCAATCATATGTACCCCCGTAAAAC
9[80]-BLK	CAAAAATAAAAGAGGACAGATGAAGAACTGAC
1[32]-BLK	TGATTCCCAAAAGGTGGCATCAATAATCATAC
17[32]-BLK	CATTAATTGGGCGCCAGGGTGGTTATTGCCCT
20[143]-BLK	AGGAGGTTTACCGTAACACTGAGTCATTCCAC
4[143]-BLK	TACCAGACCGGAATCGTCATAAATGTTCAGAA
0[143]-BLK	ACAGGTCAGGATTAGAGAGTACCTGAAGCCCG
0[47]-BLK	GCTCAACATGTTTTAAATATGCAAATAACAGT
0[63]-BLK	TGAATATAAGATTTAGTTTGACCATTTAGCTA
0[95]-BLK	TCATTTTTGCGGATGGCTTAGAGCTTAATTGC
1[112]-BLK	ATTAAGAGTTAATTGCTCCTTTTGATAAGAGG
1[128]-BLK	AAAGACTTTGACCATAAATCAAAAATAGCGTC
1[32]-BLK	TGATTCCCAAAAGGTGGCATCAATAATCATAC
1[80]-BLK	ATTTCGCAAGCAAAGCGGATTGCAGACTATTA
10[143]-BLK	TAGCCGGACCTTCATCAAGAGTAATCAACGTA
10[95]-BLK	CAACTTTGATTCGCGTCTGGCCTTAACGCCAT
11[32]-BLK	GGGATAGGTTTCCGGCACCGCTTCCATTCAGG
11[80]-BLK	CCAGTTTGCTTTGACCCCCAGCGAACACTAAA
12[143]-BLK	CTACGAAGATTTGTATCATCGCCTATGTTACT
12[47]-BLK	CAGCCAGCTCACGTTGGTGTAGATATCAACAT
12[63]-BLK	CAGGAAGATCGGTGCGGGCCTCTTGCTGCAAG
13[112]-BLK	GCAACGGCAAAGAGGCAAAAGAATTTATACCA
13[128]-BLK	CTTTGAGGTGCAGGGAGTTAAAGGACAGCTTG
13[32]-BLK	CTGCGCAAGTTTTCCCAGTCACGAATGCCTGC
14[143]-BLK	CTGAGGCTACTAAAGACTTTTTCATAATGCCA
14[95]-BLK	CAGCAGCGGGCGAAAGGGGGATGTCGCTATTA
15[32]-BLK	AGGTCGACACGAGCCGGAAGCATACTAACTCA
15[80]-BLK	TGGTCATATTATCAGCTTGCTTTCCTTTAATT
16[143]-BLK	TCACGTTGAGTTGCGCCGACAATGTTCGGTCG
16[47]-BLK	CACAACATTCTAGAGGATCCCCGGGTTGGGTA
16[63]-BLK	TCCGCTCACCGCTTTCCAGTCGGGGCCAACGC
17[112]-BLK	TTTTGCTAAGGCTCCAAAAGGAGCGAGGTGAA
17[128]-BLK	TCAACAGTCTCATAGTTAGCGTAACCAATAGG
17[32]-BLK	CATTAATTGGGCGCCAGGGTGGTTATTGCCCT
17[80]-BLK	CGTGCCAGAGTAAATGAATTTTCTCGTCTTTC

18[143]-BLK	AGACAGCCTTCAGCGGAGTGAGAATAATTTTT
18[63]-BLK	GCGGGGAGGCAGCAAGCGGTCCACTGATGGTG
18[95]-BLK	CAGACGTTCTGCATTAATGAATCGAAACCTGT
19[32]-BLK	TCACCGCCATAAATCAAAAGAATAGGAACAAG
19[80]-BLK	GCCCCAGCAGCCACCACCCTCATTGAACCGCC
2[143]-BLK	AACGAGAACAAATATCGCGTTTTAAAACTCCA
2[95]-BLK	TAGTCAGAAATGGTCAATAACCTGTTAGATAC
20[143]-BLK	AGGAGGTTTACCGTAACACTGAGTCATTCCAC
20[47]-BLK	AATCCCTTTGGCCCTGAGAGAGTTAGGCGGTT
20[63]-BLK	GTTCCGAATCCAACGTCAAAGGGCGAAAAACC
20[95]-BLK	ACCCTCAGAGGCGAAAATCCTGTTGCTGGTTT
21[112]-BLK	TTTTGCTCAGAACCGCCACCCTCATTCAGGGA
21[128]-BLK	GCGGATAAGTGCCGTCGAGAGGGTCCGTACTC
21[32]-BLK	AGTCCACTATTAAAGAACGTGGACATCGGCAA
21[80]-BLK	GTCTATCAAGAGAAGGATTAGGATTAGCGGGG
3[112]-BLK	TAGACTGGATCAGGTCTTTACCCTTCAAAAAG
3[32]-BLK	AGGCAAGGAGCCTTTATTTCAACGTTTAAATG
3[80]-BLK	AAAGCTAACAGAGGGGGTAATAGTGCAAAAGA
4[143]-BLK	TACCAGACCGGAATCGTCATAAATGTTCAGAA
4[47]-BLK	GCGGGAGACAAAGAATTAGCAAAATTTGGGGC
4[63]-BLK	ACCCTGTAAAGATTCAAAAGGGTGTATGATAT
4[95]-BLK	AGTTTTGCATCGGTTGTACCAAAACAGAGCAT
5[112]-BLK	CCACATTCATAGCGAGAGGCTTTTAAAATGTT
5[128]-BLK	CAGATACAACGTTAATAAAACGAAACTTTAAT
5[32]-BLK	CAATGCCTATGCCGGAGAGGGTAGTCATTGCC
6[143-BLK]	AAAAATCTTAACGCCAAAAGGAATCCCTCGTT
6[95]-BLK	TAGAAAGAGTCAAATCACCATCAAAGAAAGGC
7[32]-BLK	TGAGAGTCAGATTGTATAAGCAAAAATTCGCA
7[80]-BLK	TAGCATGTTAGTAAATTGGGCTTGGAAACACC
8[143-BLK]	ACAAAGCTATTACCTTATGCGATTTTGGGAAG
8[47]-BLK	AAACAGGATGGAGCAAACAAGAGATCTAGCTG
8[63]-BLK	ATCAGAAATTTTTTAACCAATAGGCCTGTAGC
9[112-BLK]	AGACCAGGAGGCTTGCCCTGACGAAGATGGTT
9[128-BLK]	CTGGCTGAACGAGGCGCAGACGGTACAAAGTA
9[32]-BLK	TTAAATTTAGCGAGTAACAACCCGACCGTAAT

Table S14. List of elongated staple strands

Designation	Sequence	Docking Sequence
9[80]-4T-V'	CAAAAATAAAAGAGGACAGATGAAGAACTGACTTTTGTGGAGTAGTGTCATGT	GTGGAGTAGTGTCATGT
Z'-4T-10[47]	AGAGTCCTAGCATATTTAGCCTTTTTAAATGTGTTGTTAAATCAGCTCAAGCCCCAA	AGAGTCCTAGCATATTTAGCC
Z'-4T-11[128]	AGAGTCCTAGCATATTTAGCCTTTTCAACGGAGGCACCAACCTAAAACGTACAGAGG	AGAGTCCTAGCATATTTAGCC
Z'-4T-14[47]	AGAGTCCTAGCATATTTAGCCTTTTACGCCAGGCTGTTGGGAAGGGCGATCGCACTC	AGAGTCCTAGCATATTTAGCC
Z'-4T-15[128]	AGAGTCCTAGCATATTTAGCCTTTTATACCGATAAAATCTCCAAAAAAAAACAACTT	AGAGTCCTAGCATATTTAGCC
Z'-4T-18[47]	AGAGTCCTAGCATATTTAGCCTTTTTGCGTATTGCGTTGCGCTCACTGCCAATTCCA	AGAGTCCTAGCATATTTAGCC
Z'-4T-19[128]	AGAGTCCTAGCATATTTAGCCTTTTAACCCATGTAGTACCGCCACCCTCAGTACCAG	AGAGTCCTAGCATATTTAGCC
Z'-4T-2[47]	AGAGTCCTAGCATATTTAGCCTTTTGCGAGCTGAATTCTGCGAACGAGTATGCTGTA	AGAGTCCTAGCATATTTAGCC
Z'-4T-3[128]	AGAGTCCTAGCATATTTAGCCTTTTCAATACTGGACGATAAAAACCAAAAACTAATG	AGAGTCCTAGCATATTTAGCC
Z'-4T-6[47]	AGAGTCCTAGCATATTTAGCCTTTTATAAATTAGAGTAATGTGTAGGTAATACTTTT	AGAGTCCTAGCATATTTAGCC
Z'-4T-7[128]	AGAGTCCTAGCATATTTAGCCTTTTCATTGTGAGCTCATTCAGTGAATACGCATAGG	AGAGTCCTAGCATATTTAGCC

Table S15. List of modified oligonucleotides

Designation	Composition	Sequence	Modification
bt-DNA	Biotin-PEG4-V	ACATGACACTACTCCAC	5'-Biotin-PEG4
bt-DNA-635P	Biotin-PEG4-V-4T-AS635P	ACATGACACTACTCCACTTTT	5'-Biotin-PEG4; 3'- AS635P
DNA-635P	9[80]-4T-V'-AS635P	CAAAAATAAAAGAGGACAGATGAAGAACTGACTTTTGTGGAGTAGTGTCATGT	3'- AS635P
AF647-DNA-bt	AF647-2T-9[80]-2T-PEG4-Biotin	TTCAAAAATAAAAGAGGACAGATGAAGAACTGACTT	5'- AF647 ; 3'- Biotin-PEG4
tetrazine-PEG5-DNA	tetrazine-PEG5-4T-V	TTTTACATGACACTACTCCAC	5'- tetrazine-PEG5
azido-PEG4-DNA	azido-PEG4-4T-V	TTTTACATGACACTACTCCAC	5'- N_3-PEG4
PNA-cysteine	O-2T-V (PNA)	O - TTACATGACACTACTCCAC	5'- O-linker
	Z-PEG4-Cholesterol	GGCTAAATATGCTAGGACTCT	3'-Cholesterol-PEG4

SI References

(1) Rothemund, P. W. K. Folding DNA to Create Nanoscale Shapes and Patterns. **2006**, *440* (March), 297–302. https://doi.org/10.1038/nature04586.

(2) Douglas, S. M.; Marblestone, A. H.; Teerapittayanon, S.; Vazquez, A.; Church, G. M.; Shih, W. M. Rapid Prototyping of 3D DNA-Origami Shapes with CaDNAno. *Nucleic Acids Res.* **2009**, *37* (15), 5001–5006. https://doi.org/10.1093/nar/gkp436.

(3) Brameshuber, M.; Weghuber, J.; Ruprecht, V.; Gombos, I.; Horváth, I.; Vigh, L.; Eckerstorfer, P.; Kiss, E.; Stockinger, H.; Schütz, G. J. Imaging of Mobile Long-Lived Nanoplatforms in the Live Cell Plasma Membrane. *J. Biol. Chem.* **2010**, *285* (53), 41765–41771. https://doi.org/10.1074/jbc.M110.182121.

(4) Brameshuber, M.; Kellner, F.; Rossboth, B. K.; Ta, H.; Alge, K.; Sevcsik, E.; Göhring, J.; Axmann, M.; Baumgart, F.; Gascoigne, N. R. J.; Davis, S. J.; Stockinger, H.; Schütz, G. J.; Huppa, J. B. Monomeric TCRs Drive T Cell Antigen Recognition. *Nat. Immunol.* **2018**, *19*, 487–496. https://doi.org/10.1038/s41590-018-0092-4.

(5) Dong, D.; Zheng, L.; Lin, J.; Zhang, B.; Zhu, Y.; Li, N.; Xie, S.; Wang, Y.; Gao, N.; Huang, Z. Structural Basis of Assembly of the Human T Cell Receptor–CD3 Complex. *Nature* **2019**, *573* (7775), 546–552. https://doi.org/10.1038/s41586-019-1537-0.

(6) Huppa, J. B.; Axmann, M.; Mörtelmaier, M. A.; Lillemeier, B. F.; Newell, E. W.; Brameshuber, M.; Klein, L. O.; Schütz, G. J.; Davis, M. M. TCR-Peptide-MHC Interactions *in Situ* Show Accelerated Kinetics and Increased Affinity. *Nature* **2010**, *463* (7283), 963–967. https://doi.org/10.1038/nature08746.

(7) Howarth, M.; Chinnapen, D. J.-F.; Gerrow, K.; Dorrestein, P. C.; Grandy, M. R.; Kelleher, N. L.; El-Husseini, A.; Ting, A. Y. A Monovalent Streptavidin with a Single Femtomolar Biotin Binding Site. *Nat. Methods* **2006**, *3* (4), 267–273. https://doi.org/10.1038/nmeth861.

(8) Fairhead, M.; Krndija, D.; Lowe, E. D.; Howarth, M. Plug-and-Play Pairing *via* Defined Divalent Streptavidins. *J. Mol. Biol.* **2014**, *426* (1), 199–214. https://doi.org/10.1016/j.jmb.2013.09.016.
